# Silent Sinus Syndrome and Williams Syndrome: Two Rare Diseases Found in a Pediatric Patient

**DOI:** 10.3389/fped.2020.00211

**Published:** 2020-04-28

**Authors:** Maddalena Petraroli, Sara Riscassi, Arianna Panigari, Marilena Maltese, Susanna Esposito

**Affiliations:** Pediatric Clinic, Department of Medicine and Surgery, Pietro Barilla Children's Hospital, University of Parma, Parma, Italy

**Keywords:** genetic disease, maxillary sinus, rare diseases, silent sinus syndrome, Williams syndrome

## Abstract

Silent sinus syndrome (SSS) is a rare disease process characterized by progressive enophthalmos and hypoglobus due to ipsilateral maxillary sinus hypoplasia and orbital floor resorption. Patients may also present with eye asymmetry, unilateral ptosis, or diplopia. Most reported cases in the literature describe its occurrence in adults, but it can also affect children. The etiology remains speculative, even though the most accepted theory is that during the first or second decade of life, occlusion of the maxillary ostium causes an interruption in normal sinus development. Williams syndrome (WS) is a rare genetic, multisystem disorder characterized by a constellation of distinctive phenotypic features, including psychomotor delay and cardiovascular abnormalities. We report a case of a 7-year-old female diagnosed at 1 year old with WS and who gradually developed SSS. This last condition was diagnosed at 7 years of age, when she started showing progressive facial asymmetry in addition to typical facial features of WS; subsequent neuroimaging definitively supported the diagnosis. This case report describes for the first time in the literature an uncommon situation in which SSS and WS, both rare syndromes, are present in the same pediatric patient. We speculate that the particular facial features typical of WS could either be the basis of the development of SSS in our patient or make the SSS clinical course more severe, with signs presenting at the age of 7 years. This case report shows for the first time that facial asymmetry in WS can be caused by SSS and highlights the need for early identification of this complication in patients with syndromes characterized by dysmorphic facial features. Further studies are needed to understand whether there is a link between the two syndromes as well as to evaluate the prevalence of SSS in patients with facial dysmorphisms and define the best management.

## Background

Silent sinus syndrome (SSS) is a rare condition characterized by progressive enophthalmos and hypoglobus due to ipsilateral maxillary sinus hypoplasia and orbital floor resorption, with or without sinonasal complaints (1). Patients may also present with eye asymmetry, unilateral ptosis, or diplopia. Most reported cases describe its occurrence in adults (2–6), but it can also affect children (1, 7). The precise pathophysiology of SSS remains speculative, even though the most accepted theory is that occlusion of the maxillary ostium causes an interruption of normal sinus development during the first or second decade of life (1, 3). Due to ostial obstruction, hypoventilation and atelectasis of the maxillary sinus cause chronic negative pressure within the sinus, leading the sinus walls to migrate inward. Bone remodeling around the maxillary sinus can be significant, leading to enophthalmos (3, 5). In SSS, the uncinate process is usually completely adherent to the lateral sinus wall, obstructing the maxillary natural ostium, although it is difficult to determine the exact causative factor leading to uncinate process displacement and ostial occlusion (1, 5). Orbitofacial imaging [computed tomography (CT) and magnetic resonance imaging (MRI) scans] is the gold standard for establishing the diagnosis. The main radiological findings described are unilateral maxillary sinus opacification and collapse, thinning and depression of the orbital floor, lateralization of the uncinate process resulting in blockage of the ostiomeatal complex and retraction of the posterolateral and medial walls of the maxillary sinus (2–6). When the condition is left untreated, it may result in complete obliteration of the sinus with worsening enophthalmos and hypoglobus. Functional endoscopic sinus surgery with uncinectomy and middle meatal antrostomy, in order to relieve the blockage of the ostiomeatal complex, should be the treatment of choice in patients presenting with enophthalmos and/or hypoglobus, even in children (1, 7). The reconstruction of the orbital floor should be considered only as a second stage procedure in those cases with relevant aesthetic deformities or persistence of orbital symptoms (1).

Williams syndrome (WS), also referred to as Williams-Beuren syndrome, is a rare genetic, multisystem disorder with an estimated prevalence of 1:10,000 live births. Affected people may have typical facial features, cardiovascular anomalies, growth failure, skeletal abnormalities, hypercalcemia, endocrine disorders, and a distinct neurodevelopmental and behavioral profile (8–11). WS is caused by a variably sized hemizygous deletion within band 11.23 of the long arm of chromosome 7, involving 26–28 genes. It includes the *ELN* gene, which codes for the protein elastin and is responsible for some of the typical phenotypic findings in patients with WS, such as facial features, a hoarse voice, cardiovascular disease, bladder and bowel diverticula, and orthopedic problems (9, 10). Most children with WS are described as having similar facial features, which are often subtle in the first years of life but tend to become more distinctive with advancing age; they often include periorbital fullness, a short nose with a bulbous nasal tip, a long philtrum, a wide mouth, full lips, and mild micrognathia (8). Other medical problems in patients with WS include hypodontia, malocclusion, joint laxity and joint contractures (9, 10). Patients with WS could have facial asymmetry but without related symptoms (8).

We report a case of a 7-year-old girl affected by WS who subsequently developed SSS. Currently, there are no reported cases that describe the association between WS and SSS.

## Case Presentation

A 7-year-old female affected by WS was genetically diagnosed at 1 year of age [deletion of region 11.23 on the long arm of chromosome 7 confirmed by dual color fluorescent *in situ* hybridization (FISH)]. She was born by spontaneous delivery at 41 weeks of gestation after a regular pregnancy. There was no family history of significant disease or anomalies. Our patient was followed-up for pulmonary artery stenosis, behavioral problems, intellectual disability, Achilles tendinopathy, and early puberty.

**Figure 1:**
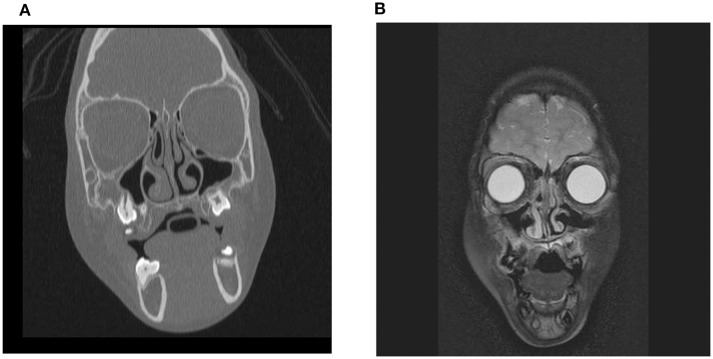
Facial MRI **(A)** and CT **(B)** scans performed in the patient with WS when she was 5 years old. No significant asymmetry between the maxillary sinuses was observed.

On clinical examination, she had dysmorphic facial features that are often seen in WS: periorbital fullness, a short nose with a bulbous nasal tip, a long philtrum, a wide mouth and full lips. Facial MRI (Figure 1A) and CT (Figure 1B) performed when she was 5 years old for the evaluation of dysmorphic facial features did not show any significant asymmetry between the maxillary sinuses.

**Figure 2:**
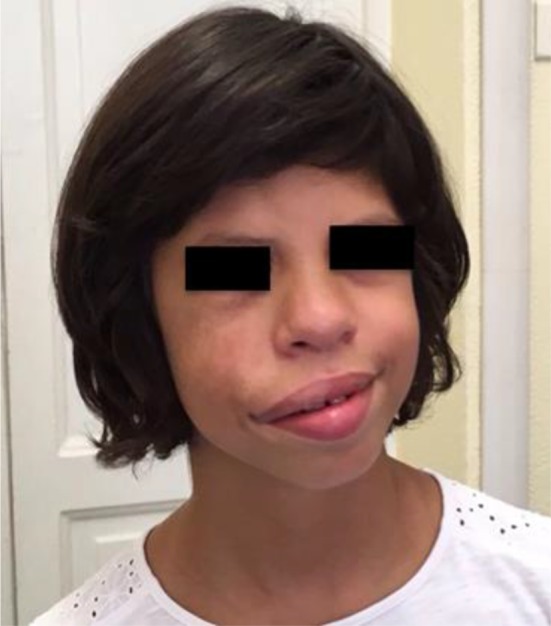
The patient at the age of 7 years. the physical examination showed progressive facial asymmetry with right hypoglobus and enophthalmos that had never been reported during her first years of life.

During her follow-up, at 7 years of age the physical examination showed progressive facial asymmetry with right hypoglobus (i.e., inferior displacement of the globe in the orbit) and enophthalmos that had never been reported during the first years of life (Figure 2). The patient had neither a history of sinonasal complaints nor recurrence of upper respiratory tract infections. Moreover, the patient did not complain of visual disturbances or facial pain.

**Figure 3:**
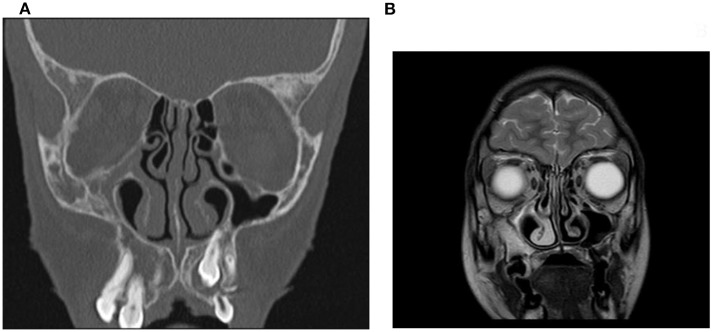
Facial MRI **(A)** and CT **(B)** scans performed in the patient with WS when she was 7 years old. MRI showed that the right maxillary sinus was opacified, with central inspissated secretions; it was also diminished in volume with inward bowing of the maxillary walls and the uncinate process was lateralized **(A)**. CT scan confirmed the opacified right maxillary sinus with retraction of the maxillary walls as well as the lateralized and demineralized uncinate; it also demonstrated the partial occlusion of the maxillary infundibulum and the fact that the orbital floor was located inferiorly.

However, at 7 years old, she developed early puberty and underwent skull base imaging to study the sellar region and start treatment with gonadotrophin releasing hormone (GnRH) agonists. This imaging showed a constellation of radiological features typical of SSS (Figure 3). Facial MR scan revealed a hypoplastic and opacified right maxillary sinus with retraction of the maxillary sinus walls and diminished sinus volume (Figure 3A). To support the evidence from MR imaging, a CT scan was performed and showed a diminished volume of the right maxillary sinus, which was completely opacified; the CT scan also showed a lateralized uncinate process with retraction of the middle turbinate and demineralization of the sinus walls (Figure 3B). These radiological findings led to the diagnosis of SSS.

At the time of writing, the patient is 9 years old and her enophthalmos and facial asymmetry looked stable. Endoscopic sinus surgery, which is the treatment of choice, has not been performed but it is considered in case of worsening.

## Discussion

This case report describes, for the first time, that facial asymmetry in WS can be caused by SSS. SSS is a rare clinical condition characterized by progressive enophthalmos, hypoglobus and asymmetry of the eyes secondary to maxillary sinus hypoplasia and orbital floor resorption, generally affecting patients in their third to fifth decade of life (1). No data are available regarding SSS prevalence in pediatric patients. WS is a rare genetic, multisystem disorder characterized by typical facial features, hypodontia and malocclusion, cardiovascular anomalies, growth failure, skeletal abnormalities, hypercalcemia, endocrine disorders, and a distinct neurodevelopmental and behavioral profile (7–10).

We speculate that the particular facial features typical of WS could either be the basis of the development of SSS in our patient or make the SSS clinical course more severe, with signs presenting at the age of 7 years. Ultimately, this association could be random; however, considering the rarity of these two syndromes, it is truly curious and challenging to possess them simultaneously. The method used for WS diagnosis (i.e., FISH) does not allow a more in-depth analysis of the deleted genes in the patient in question. If genomic hybridization (CGH) or whole exome sequencing had been used, we could try to make a causative relationship. Further studies are needed to understand whether there is a link between the two syndromes as well as to evaluate the prevalence of SSS in patients with facial dysmorphisms and define the best management.

## Conclusions

This case report shows for the first time that SSS could occur in children with WS and possibly explain the facial asymmetry. Although the association of the two disorders could be without direct causative relationship and we did not explore the genetic basis behind this, it highlights the need for early identification of this complication in patients with syndromes characterized by dysmorphic facial features.

## Data Availability Statement

All datasets generated for this study are included in the article/supplementary material.

## Ethics Statement

The studies involving human participants and the publication of this case report were reviewed and approved by Area Vasta Emilia Romagna Nord. Written informed consent to participate in this study was provided by the participants' legal guardian/next of kin. Written informed consent was obtained from the individual(s), and minor(s)' legal guardian/next of kin, for the publication of any potentially identifiable images or data included in this article, including photography.

## Author Contributions

MP performed the diagnosis and was in charge of the patient's follow-up. SR and AP wrote the first draft of the manuscript. MM performed the literature review. SE provided scientific contributions and critically revised the paper. All of the authors have read and approved the final version of the manuscript.

## Conflict of Interest

The authors declare that the research was conducted in the absence of any commercial or financial relationships that could be construed as a potential conflict of interest.
